# Public health round-up

**DOI:** 10.2471/BLT.16.010116

**Published:** 2016-01-01

**The joy of ageing**

A new World Health Organization (WHO) campaign on Instagram is encouraging people to share images celebrating the lives of people at older ages, such as this photograph of retired midwife, Lesley, who helped to bring her granddaughter, Isla, into the world. Instagram is a social networking service that allows users to share pictures and videos. Photos can be submitted on Instagram or Twitter using the hashtag #YearsAhead, or sent to healthyageing@who.int with the subject line “YearsAhead”.

http://www.who.int/ageing/features/yearsahead-campaign/en/

**Figure:**
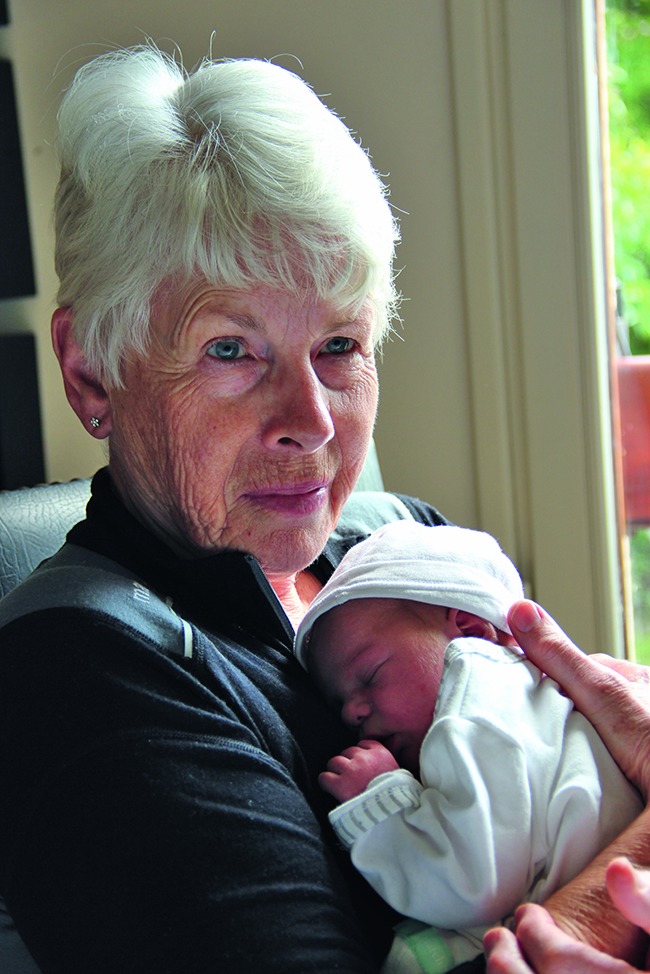
WHO/A. Officer

## HIV spike in European Region

The highest number of new HIV cases since reporting began in the 1980s was recorded in WHO’s European Region in 2014, according to the European Centre for Disease Prevention and Control (ECDC) and the WHO Regional Office for Europe.

WHO’s 53-country European Region stretches from Portugal in the west all the way across the continent to Kazakhstan in the east.

“Despite all the efforts to fight HIV, this year the European Region has reached over 142 000 new HIV infections, the highest number ever. This is a serious concern,” says Dr Zsuzsanna Jakab, WHO Regional Director for Europe.

“With all the evidence on HIV prevention and control, including new treatment guidelines, we call on European countries to take bold action and curb the HIV epidemic once and for all,” she said, referring to WHO’s consolidated guidelines that provide advice on many aspects of treatment and care for people with HIV infection.

The growth of the HIV epidemic was driven by countries in the eastern part of the region, where the number of new diagnoses has more than doubled during the past decade, the ECDC and WHO said.

Heterosexual transmission of HIV infection was responsible for the increase in eastern European countries, while sex between men was the predominant mode of HIV transmission in Western Europe.

Last month WHO called on all health decision-makers to ensure that everyone living with HIV has access to treatment with antiretroviral medicines.

http://www.euro.who.int/en/media-centre/sections/press-releases/2015/11/highest-number-of-new-hiv-cases-in-europe-ever

## Foodborne diseases

Foodborne diseases kill an estimated 125 000 children under the age of five every year, according to a WHO report. This means that almost one third (30%) of all deaths from contaminated food are in small children, even though this age group represents only 9% of the global population.

The new report, entitled *Estimates of the global burden of foodborne diseases*, is the most comprehensive report of its kind to date and presents the latest data on illnesses caused by 31 disease-causing agents, including bacteria, viruses, parasites, chemicals and toxins.

According to the new figures released last month, an estimated 600 million people become ill after eating contaminated food and 420 000 of those people died of the consequences.

“Until now, estimates of foodborne diseases were vague and imprecise. This concealed the true human costs of contaminated food. This report sets the record straight,” said Dr Margaret Chan, Director-General of WHO.

More than half of the global burden of foodborne diseases is attributed to diarrhoeal diseases.

These are commonly caused by the consumption of undercooked or raw eggs, meat and dairy products – food products that have become contaminated by norovirus, *Campylobacter*, non-typhoidal *Salmonella* and pathogenic *Escherichia coli*.

Children are worst affected, with 220 000 children falling ill and 96 000 dying every year as a result of foodborne diarrhoeal diseases.

People from low- and middle-income countries are at higher risk as foodborne diseases are linked to food preparation with unsafe water, poor hygiene patterns and inadequate food storage. Lower levels of literacy and education and insufficient food safety systems are also contributory factors.

The WHO report highlights the need for governments, food manufacturers and citizens to take action to prevent foodborne diseases. WHO is working with governments to help them to design and implement more efficient food-safety policies.

http://www.who.int/foodsafety/publications/foodborne_disease/fergreport/en/

**Cover photo**

Health workers providing the oral polio vaccine at Torkhan on the Afghanistan border during the National Immunization Campaign in 2014. About 1.3 million oral vaccinations are administered every year to children at the Torkham Gate crossing, the focal point of an intense global campaign to eradicate polio by 2018.

**Figure:**
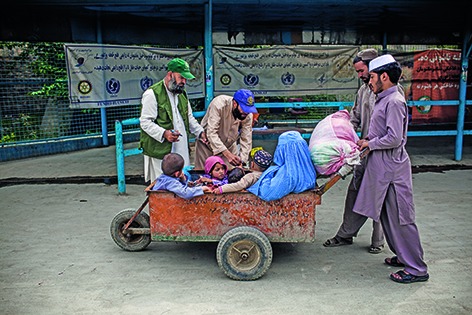
© Diego Ibarra Sánchez / MeMo

## Sexual violence: guiding the response

A new WHO and the United Nations Office on Drugs and Crime (UNODC) guide provides advice on how to document cases of sexual violence. 

The guide provides advice for health care providers, forensic specialists, police, lawyers and judges on how to collect evidence and carry out a forensic examination. A good medico-legal response is an important element in ensuring that perpetrators can be brought to justice. 

While several governmental and nongovernmental organizations are working to address sexual violence in many countries, the response to this problem often lacks the medical-legal knowledge and coordination needed to bring the perpetrators to justice. 

The guidance entitled *Strengthening the medico-legal response to sexual violence* is the result of a joint project between WHO and the United Nations Office on Drugs and Crime. 

The project is supported by the United Nations Action against Sexual Violence in Conflict – a network of 13 United Nations agencies, spanning humanitarian, technical, political and peacekeeping, that work together to strengthen the response to conflict-related sexual violence.

The guide is presented in the form of one-page reference cards, containing facts about sexual violence, and advice on how to take a statement or conduct a forensic examination, how to provide care, and how to strengthen coordination across the multiple actors that are involved in the medico-legal response.

In 2008, the United Nations Security Council adopted resolution SCR 1820, calling on all armed conflict parties to stop acts of sexual violence. Several other resolutions have since addressed the issue, most recently Security Council resolution 2106 in 2013 that reiterates the demand for complete cessation of all acts of sexual violence by all parties to armed conflicts. 

http://www.who.int/reproductivehealth/topics/violence/medico-legal-response/en/

## Health at the Paris summit

How can countries ensure that their actions to cut greenhouse gas emissions also reduce the huge disease burden from air pollution?

Which population groups are likely to be affected by the health impacts resulting from climate change, of diarrhoeal and vector-borne diseases, undernutrition and extreme weather?

These and other questions were discussed at a WHO side-event at the United Nations climate change conference – the Conference of the Parties (COP 21) to the United Nations Framework Convention on Climate Change Convention (UNFCCC) – in Paris last month.

Climate talks in the past rarely referred to human health or the health benefits of taking action to reduce carbon dioxide emissions. Awareness of these health aspects have increased in recent years.

Health is included in two key articles of the UNFCCC and WHO is working with the UNFCCC secretariat to provide support to countries in designing the health component of national plans for adapting to climate change.

The side-event on 8 December aimed to raise the profile of health at the Paris summit. It was organized by WHO in collaboration with other United Nations agencies and the Council on Biodiversity with support from the French Ministry of Health.

WHO co-sponsored and collaborated on several other events at the summit on themes related to health adaptation to climate change, the health benefits of mitigating climate change, and the links between health, air pollution and climate.

http://www.who.int/globalchange/global-campaign/en/

## Malaria vaccine

Pilot studies of the world’s first malaria vaccine are needed to find out the best way to deliver the vaccine, according to a group of experts.

The vaccine, called RTS,S/AS01, is given as a four dose schedule, starting at 5 months of age. The first three doses are given every 30 days, followed by a fourth dose 18 months later.

The Strategic Advisory Group of Experts on Immunization (SAGE) and the Malaria Policy Advisory Committee – which advise WHO – met in October to discuss how such a four-dose vaccination schedule could be integrated into child immunization programmes.

The two committees agreed that pilot implementation projects in three to five sub-Saharan African countries should be the next step to see how the vaccine might best be delivered. 

WHO has adopted these recommendations and is strongly supportive of the need to proceed.

The vaccine is effective against *Plasmodium falciparum*, the most prevalent malaria parasite in Africa and the most lethal malaria parasite worldwide, and is being assessed as a complement to the current recommended malaria control package of insecticide-treated bed nets, rapid diagnostic tests and medicines to prevent and treat malaria.

In July, after considering the findings of clinical trials of the vaccine, the European Medicines Agency gave a positive opinion on the quality as well as the risks and benefits of the vaccine. The manufacturer, GlaxoSmithKline, has yet to make regulatory submissions to African countries.

Currently there are no licensed vaccines against malaria or any other human parasite. To date, no vaccine has ever been licensed against a parasitic disease in humans.

http://www.who.int/immunization/research/development/malaria_vaccine_qa/en/

**Looking ahead**

25–30 January – Executive Board 138th session. Geneva, Switzerland.

7 April – World Health Day. The 2016 theme is diabetes.

23–28 May – Sixty-ninth World Health Assembly, Geneva, Switzerland.

